# MYC-Associated Factor MAX is a Regulator of the Circadian Clock

**DOI:** 10.3390/ijms21072294

**Published:** 2020-03-26

**Authors:** Olga Blaževitš, Nityanand Bolshette, Donatella Vecchio, Ana Guijarro, Ottavio Croci, Stefano Campaner, Benedetto Grimaldi

**Affiliations:** 1 Molecular Medicine Research Line, Fondazione Istituto Italiano di Tecnologia (IIT), 16135 Genoa, Italy; olga.blazevits@iit.it (O.B.); Nityanand.Bolshette@iit.it (N.B.); donatella.vecchio@iit.it (D.V.); anaguijarroa@yahoo.es (A.G.); 2 Center for Genomic Science, Fondazione Istituto Italiano di Tecnologia (IIT), 20139 Milan, Italy; ottavio.croci@iit.it (O.C.); stefano.campaner@iit.it (S.C.)

**Keywords:** circadian clock, MYC-associated factor X, clock network, MAX network, MNT, BMAL1, cancer, MYC

## Abstract

The circadian transcriptional network is based on a competition between transcriptional activator and repressor complexes regulating the rhythmic expression of clock-controlled genes. We show here that the MYC-associated factor X, MAX, plays a repressive role in this network and operates through a MYC-independent binding to E-box-containing regulatory regions within the promoters of circadian BMAL1 targets. We further show that this “clock” function of MAX is required for maintaining a proper circadian rhythm and that MAX and BMAL1 contribute to two temporally alternating transcriptional complexes on clock-regulated promoters. We also identified MAX network transcriptional repressor, MNT, as a fundamental partner of MAX-mediated circadian regulation. Collectively, our data indicate that MAX regulates clock gene expression and contributes to keeping the balance between positive and negative elements of the molecular clock machinery.

## 1. Introduction

Many cellular processes are regulated by an endogenous cell-autonomous clock (the circadian clock) that has an intrinsic period of approximately 24 h [1,2,3]. The molecular mechanism underlying these circadian rhythms is based on the interconnected transcriptional–translational feedback loops where specific transcription factors repress the expression of their own target genes [4,5,6].

Studies in cultured cells clearly showed the cell-autonomous feature of the transcriptional circadian rhythmicity and allowed the dissection of the molecular architecture of the clock [7,8].

Accordingly, the clock core network has been conceptualized as two transcriptional complexes operating in an antagonistic manner on the expression of clock-controlled genes (CCGs). On the one hand, the proteins CLOCK and BMAL1 interact to form a clock activator complex that stimulates the transcription of CCGs by recognizing E-box and E-box-like cis-regulative elements proximal to their core promoters [9]. On the other hand, the association of PERIOD and CRYPTOCHROME proteins with CLOCK/BMAL1 forms a transcriptional repressor complex that decreases CLOCK/BMAL1-dependent transcription [10,11,12,13]. The periodic competition between clock-activator and clock-repressor complexes determines the circadian expression of around 5%–10% of the mammalian transcriptome [14,15,16]. Nevertheless, additional negative regulators, such as REV-ERB nuclear receptors [13,17], appear important for a proper circadian rhythm, and mathematical modelling of the circadian clock gene-regulatory network indicated that a synergy of multiple inhibitions are required for robust self-sustained oscillations [18]. In addition, a proper balance between activators and repressors of E-boxes has been proposed as a crucial requirement for generating circadian rhythms [19,20].

Disruption of the molecular clock is associated with a variety of human pathologies, including cancer [6,21]. Ectopic overexpression of the oncogenic MYC protein has been recently reported to alter circadian gene expression in cancer cell lines, although the molecular mechanism behind this MYC function is still highly debated [22,23,24,25].

MYC is a transcription factor that can either activate or repress transcription depending on the interacting protein partners [26,27]. As a heterodimer with the MYC-associated X-factor (MAX) protein, MYC stimulates transcription of diverse genes bearing promoter-proximal E-boxes, including important cell cycle and metabolic genes. However, MYC can also repress gene expression when recruited in complex with MIZ1 to non-E-box sites in the promoters of MIZ1 target genes [28,29,30].

Whether MYC-mediated alteration of the circadian rhythm depends on one or both mechanisms is still debated. Indeed, MYC overexpression was shown either to interfere with E-box-driven transcription of the BMAL1-containing molecular clock complex [22] or to act as a direct transcriptional repressor of BMAL1 in an E-box-independent fashion [23].

We report here that MAX operates as an unexpected integral regulator of the circadian transcriptional network in an MYC-independent manner in both cancer and non-cancerous cell lines. We further identified the MAX-binding protein MNT as a fundamental component of MAX-mediated clock regulation.

## 2. Results

### 2.1. Knockdown of MAX Represses the Transcription of Core Clock Genes in Cancer Cell Lines

Studies in U2OS cells overexpressing an ectopic MYC protein have shown that elevated levels of MYC can profoundly alter the expression of CLOCK/BMAL1-regulated genes [22,23]. Upregulation of MYC has been reported in many triple-negative breast cancer (TNBC) cells [31]. Accordingly, we observed elevated levels of MYC protein in a TNBC cell line, MDA-MB-231, compared with non-cancerous MFC10A and non-triple negative breast cancer cells (Supplementary Figure S1A).

**Figure 1:**
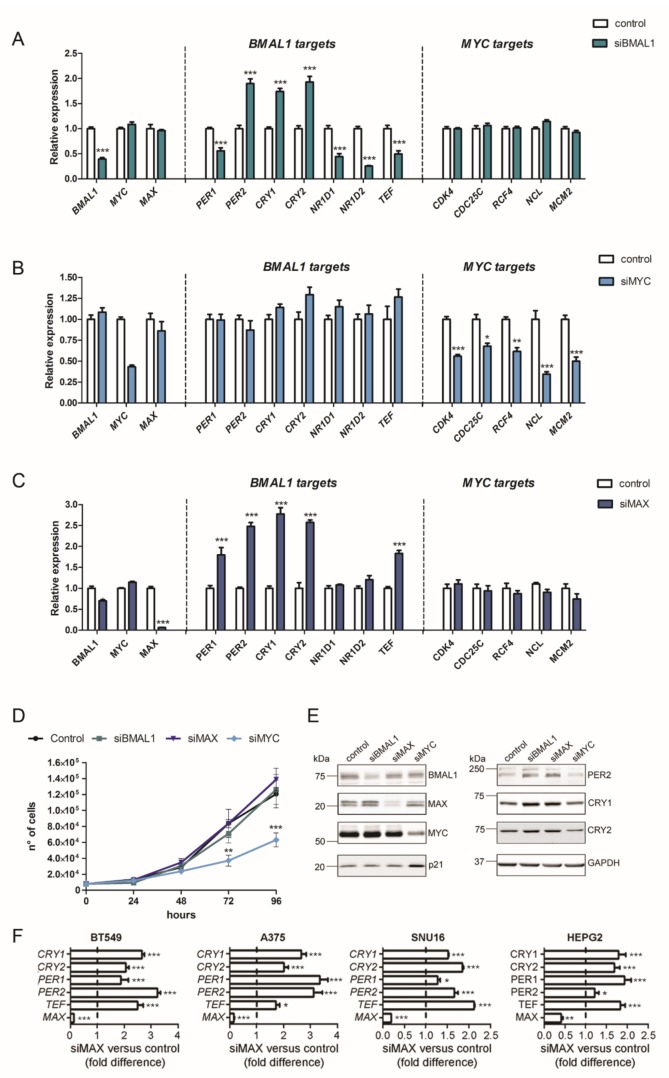
Knockdown of *MAX* alters the expression of core clock genes. (**A**–**C**) Expression of the circadian BMAL1 targets and the cell cycle MYC targets in MDA-MB-231 with knocked down *BMAL1* (siBMAL1), *MYC* (siMYC), or *MAX* (siMAX). A non-coding siRNA was used as control. Relative expression was determined by qRT–PCR using *GAPDH* for normalization. The values of control cells were set to 1. Shown as mean ± SEM, *n* ≥ 6. * *p* < 0.05. ** *p* < 0.01 and *** *p* < 0.001, two-way ANOVA with Bonferroni post hoc test, silencing versus control. (**D**) Growth curve of MDA-MB-231 transfected with siRNA sequences against *BMAL1*, *MYC*, *MAX*, or a non-targeting control. Shown as mean ± SEM, *n* ≥ 6. ** *p* < 0.01 and *** *p* < 0.001, two-way ANOVA with Bonferroni post hoc test, siMYC versus control. (**E**) Immunoblot of protein samples from siBMAL1, siMYC, siMAX, and control MDA-MB-231 cells with specific antibodies against the indicated proteins. GAPDH was used as a loading control. (**F**) The expression of *MAX*, *CRY1*, *CRY2*, *PER1*, *PER2*, and *TEF* was analyzed in breast (BT549), skin (A375), stomach (SNU16), and liver (HEPG2) cancer cell lines knocked down for *MAX*. The values of control cells were set to 1 (dotted line). Shown as mean ± SEM, *n* = 3. * *p* < 0.05 and *** *p* < 0.001, two-way ANOVA with Bonferroni post hoc test, siMAX versus control.

We thus decided to evaluate whether MYC/MAX complex might control clock gene transcription in MDA-MB-231 by comparing the mRNA levels of core clock genes in cells in which expression of *BMAL1*, *MYC,* or *MAX* were knocked down by siRNA. In line with observations in *Bmal1^−/−^* mice [32,33] and with direct and indirect roles of BMAL1 in regulating clock-controlled genes [32,34,35], cells knocked down for *BMAL1* differently altered the expression of diverse clock-controlled genes (*PER1*, *PER2, NR1D1*, *NR1D2*, *TEF, CRY1,* and *CRY2*) (Figure 1A). Conversely, the expression of characterized MYC target genes involved in cell proliferation [36], such as *CDK4*, *CDC25C*, *RCF4*, *NCL,* and *MCM2*, showed negligible differences in *BMAL1*-silenced cells compared with control cells (Figure 1A).

While knockdown of *MYC* markedly reduced mRNA levels of MYC proliferative-related targets, it had negligible effects on BMAL1-regulated genes (Figure 1B). Strikingly, cells with knocked down *MAX* showed drastic alterations in diverse clock transcripts (Figure 1C). Indeed, diverse core clock repressor genes were significantly upregulated upon MAX silencing (*PER1*, *PER2*, *CRY1,* and *CRY2*), while BMAL1 expression was reduced (Figure 1C).

Notably, the decline of MAX in *MAX*-silenced cells was not sufficient for significantly influencing proliferative MYC targets, suggesting that the remaining MAX protein could still ensure a proper function of the MAX/MYC complex.

In line with our transcriptional data, knockdown of either *MAX* or *BMAL1* had no effect on cell proliferation, while *MYC*-silenced MDA-MB-231 cells showed significantly reduced growth compared with control cells (Figure 1D). Immunoblot analysis in MDA-MB-231-silenced cells confirmed that reduction of either BMAL1- or MAX-elevated PER2, CRY1, and CRY2 protein levels, whereas knockdown of MYC had no such effect (Figure 1E). Consistent with the observation that only *MYC*-silencing influenced MDA-MB-231 proliferation, protein levels of the cell cycle regulator cyclin dependent kinase inhibitor 1A (CDKN1A, also known as p21) showed differences solely in *MYC*-silenced cells (Figure 1E).

Knockdown of *MAX* expression by using two additional diverse and non-redundant siRNA sequences against *MAX* transcripts similarly affected *BMAL1*, *PER1*, *PER2*, *CRY1*, *CRY2*, and *TEF* transcript levels (Supplementary Figure S1B), thus ruling out that altered clock gene expression in *MAX*-silenced cells derived from off-target effects.

Notably, the knockdown of MAX significantly increased the expression of clock genes in a different TNBC cell line, BT549, as well as in cancer cell lines originated from skin (A375), stomach (SNU16), and liver (HEPG2) tumors (Figure 1F), thus indicating that MAX-mediated regulation of clock genes might be extended to diverse human cell lines.

### 2.2. MAX-Inhibition of the Core Clock Genes is Independent of CRY-Mediated Repression but Requires a Functional E-Box Responsive Element

**Figure 2:**
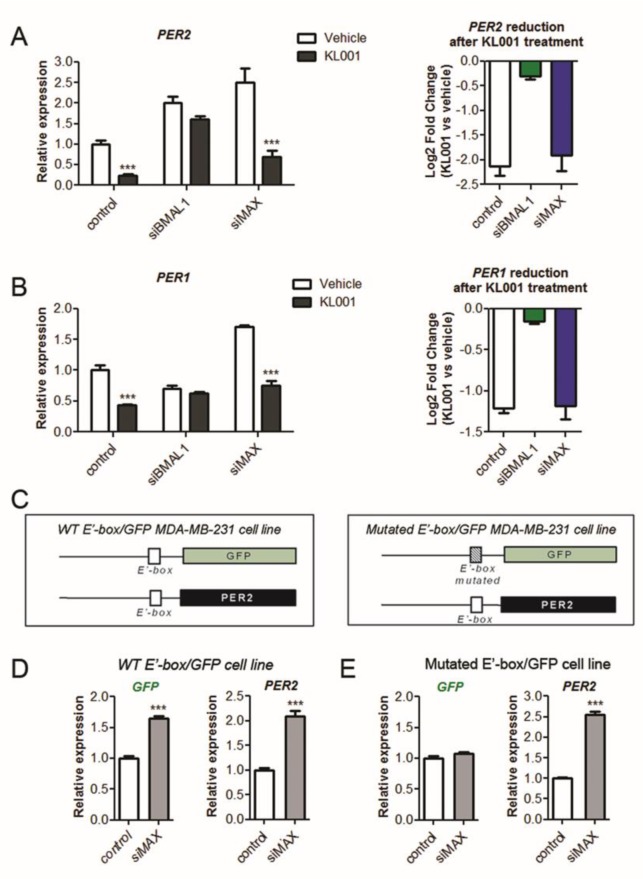
MAX-inhibition of the core clock genes is independent of CRY-mediated repression but requires a functional E-box responsive element. (**A**,**B**) MDA-MB-231 cells transfected with siRNA sequences against *BMAL1*, *MAX,* or a non-targeting control were treated 24 h with vehicle (DMSO) or 10 µM CRY1 agonist (KL001). The effect of KL001 on the expression of CRY1 targets, *PER1* and *PER2*, was evaluated by qRT–PCR using *GAPDH* for normalization. The values of untreated control cells were set to 1. Shown as mean ± SEM, *n* = 3. *** *p* < 0.001, two-way ANOVA with Bonferroni post hoc test, vehicle versus KL001. Right panel reports the log2 ratio between KL001 and vehicle samples, thus showing reduction of *PER1* or *PER2* expression after the treatment with CRY agonist. (**C**) Schematic representation of two MDA-MB-231 reporter cell lines bearing the sequence coding for the green fluorescent protein (GFP) gene controlled by a promoter fragment of *PER2* containing a wild-type or a mutated version of the clock regulated E’-box element (*WT E’-box/GFP* and *Mutated E’-box/GFP* cells, respectively). (**D**,**E**) Expression of the endogenous *PER2* gene and the GFP is driven by a *WT E’-box* or a *mutated E’-box* in reporter cells with knocked down *MAX* (siMAX). A non-coding siRNA was used as control. Relative expression was determined by qRT–PCR using *GAPDH* for normalization. The values of control cells were set to 1. Shown as mean ± SEM, *n* = 4. *** *p* < 0.001, two-tailed Student’s *t*-test, MAX silencing versus control.

The effect of MAX silencing on the transcription of clock genes resembles the molecular phenotype observed in cells or tissues lacking CRY repressor proteins (i.e., transcriptional de-repression of CLOCK/BMAL1/CRY targets) [5,32]. We thus investigated whether MAX might affect CRY-mediated repression by evaluating the expression of *PER1* and *PER2* following treatment with a CRY agonist (KL001 [37]) in MDA-MB-231 knocked down for *BMAL1* or *MAX*. As expected, KL001-augmented CRY transcriptional repression in control cells, as indicated by the significant decrease of *PER1* and *PER2* mRNA levels in KL001-treated cells compared with a vehicle (Figure 2A,B). In line with the essential role of CLOCK/BMAL1 complex in mediating CRY1 activity [5], the knockdown of *BMAL1* strongly reduced KL001-mediated inhibition of *PER1* and *PER2* transcription.

In contrast, KL001 efficacy was preserved upon *MAX* knocked down, as indicated by a comparable drug-related decrease in *PER* transcripts in both *MAX*-silenced and control cells (Figure 2A,B). In addition, *MAX* silencing significantly enhanced the transcription of *PER1, PER2, CRY2*, and *TEF* in both control and *CRY1*-silenced cells (Supplementary Figure S2A). Collectively, our results suggest that MAX repression operates independently from the activity of the BMAL1/CLOCK/CRY repressor complex.

Considering that MAX directly interacts with E-box and E-box-like elements [38,39], we then evaluated whether this transcription factor regulates the expression of clock target genes by acting on clock-responsive E-box regulatory regions. To evaluate this aspect, we generated two transgenic MDA-MB-231 cell lines expressing the green fluorescent protein (*GFP*) gene under the control of a promoter fragment of *PER2* containing either a wild-type or a mutated E’-box element (*E’-box-GFP and E’^mut^-box-GFP* cells, respectively; Figure 2C). Indicating a functional clock regulation of our cell-based reporter system, KL001 reduced the expression of *GFP* in *E’-box-GFP*, but not in *E’^mut^-box-GFP* cells (Supplementary Figure S2B). As an internal control, KL001 treatment inhibited the transcription of the endogenous *PER2* gene in both cell lines.

We thus evaluated the effect of MAX silencing in the GFP reporter cells. Revealing that MAX requires a functional E’-box for its transcriptional activity, the knockdown of *MAX* reduced GFP expression in *E’-box-GFP*, but not in *E’-box^Mut^-GFP* cells (Figure 2D,E). In contrast, endogenous *PER2* transcription was elevated in both cell lines upon the silencing of either *BMAL1* or *MAX*.

### 2.3. MAX is Recruited on BMAL1 Bound Genomic Regions in a MYC-Independent Manner

**Figure 3:**
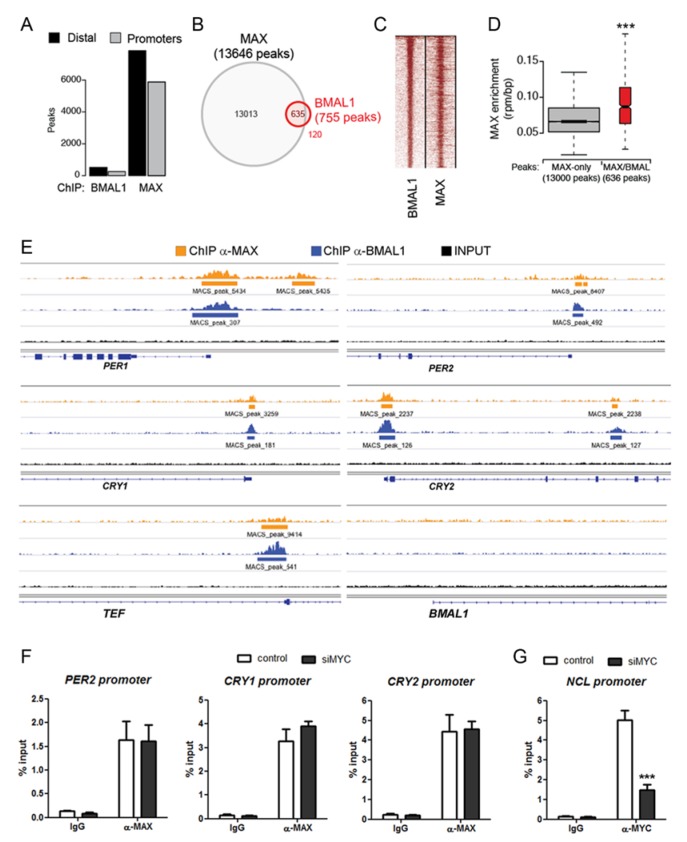
MAX is recruited on BMAL1-bound genomic regions. (**A**) Number of peaks identified for ChIP-seq of BMAL1 and MAX in MDA-MB-231 cells. Sub-setting of distal or promoter peaks was based on their proximity to annotated transcriptional starting site. (**B**) Venn diagram showing the overlap of ChIP-seq peaks of BMAL1 and MAX. (**C**) Heatmap of ChIP-seq signals of genomic regions co-bound by BMAL1 and MAX showing the signals for both TF factors on shared regions and their relative intensities. (**D**) Box-plot showing the enrichment of MAX in genomic regions bound by MAX (MAX only) or by both MAX and BMAL1 (MAX/BMAL1). *** *p*< 0.001, two-tailed Student *t*-test. (**E**) Genomic snapshots of the promoter region of core circadian clock genes (*PER1*, *PER2*, *CRY1*, *CRY2*, *TEF*, *BMAL1*) showing the enrichment of MAX (orange) and BMAL1 (blue). Of note, all these regions contain either E-box or E’-box sequences. (**F**) Recruitment of MAX on the E-box-containing promoters of PER2, CRY1, and CRY2 in MYC-silenced and control MDA-MB-231 cells. Enrichment of MAX was evaluated by quantitative PCR of immunoprecipitated DNA compared with input DNA (% of input). Immunoglobulin G (IgG) was used as a negative control. Shown as mean + SEM, *n* =3. (**G**) Chromatin samples from (**F**) were immunoprecipitated with α-MYC antibody to confirm the actual reduction of MYC recruitment on an MYC-target gene (NCL) in MYC-silenced cells. *** *p* < 0.001, two-tailed Student *t*-test.

Our results with the GFP-reporter cell lines suggest that BMAL1 and MAX might be recruited on the same E-box containing regulatory regions within the promoters of clock target genes. To explore this possibility, we performed chromatin immuno-precipitation sequencing (ChIP-seq) experiments with specific antibodies against BMAL1 and MAX proteins. This analysis revealed a large number of genomic regions bound by MAX (around 13,000 peaks), while BMAL1 binding was limited to about 800 regions (Figure 3A; Supplementary Tables S1 and S2). BMAL1 and MAX bound regions comprised both promoters and distal sites. Coherently with the circadian role of BMAL1 and its preference for E-box-containing sites, ontological annotation of BMAL1 bound regions showed significant enrichment for circadian regulated genes and E-box motifs (Supplementary Figure S3). Remarkably, 85% of the BMAL1 bound sites overlapped with MAX bound regions (Figure 3B) and heatmap visualization of the ChIP-seq signals on the peaks shared by BMAL1 and MAX showed a robust enrichment on these regions for both transcription factors (Figure 3C). Furthermore, the enrichment of MAX was significantly higher on the genomic regions bound by BMAL1 than on the loci sites in which BMAL1 was not present (Figure 3D).

Strikingly, MAX was present on the E-box-containing regions of all the circadian factors upregulated upon MAX silencing (*PER1*, *PER2*, *CRY1*, *CRY2*, and *TEF*; Figure 3E), supporting a direct transcriptional repressive activity of MAX on the clock molecular machinery.

We further evaluated whether the recruitment of MAX on BMAL1 target promoters might be independent of MYC by immunoprecipitation of MYC-silenced and control chromatin samples with an anti-MAX antibody. Strikingly, MYC silencing resulted in negligible differences in the enrichment of MAX on the promoters of *PER2*, *CRY1,* and *CRY2* (Figure 3F). Confirming the actual reduction of MYC in *MYC*-silenced cells, ChIP with an anti-MYC antibody showed a drastic reduction of MYC recruitment on the *NCL* promoter in cells knocked down for *MYC*, compared with control (Figure 3G).

**Figure 4:**
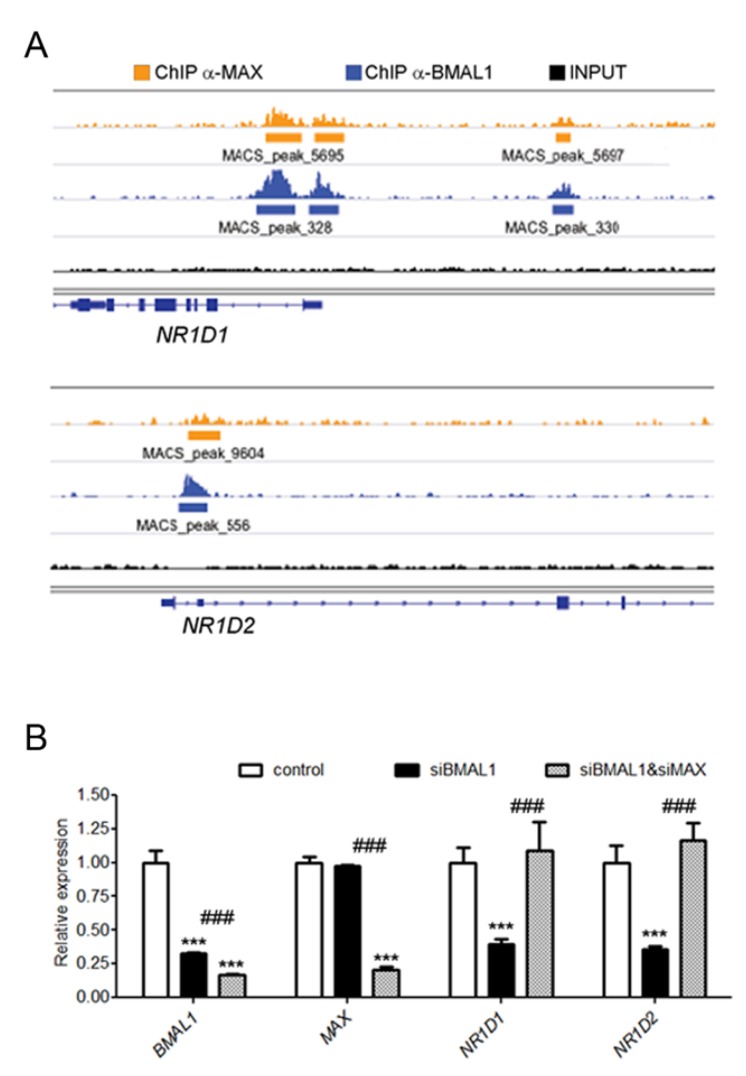
MAX regulates REV-ERB expression. (**A**) Genomic snapshots of the promoter region of the circadian repressor genes, *NR1D1* (also known as *REV-ERBα*) and *NR1D2* (also known as *REV-ERBβ*) showing the enrichment of MAX (orange) and BMAL1 (blue) ChIP-seq signals. (**B**) Expression of *NR1D1* and *NR1D2* in MDA-MB-231 cells upon the knockdown of either *BMAL1* (siBMAL1) or *BMAL1* and *MAX* (siBMAL1 and siMAX). Relative expression was determined by qRT–PCR using *GAPDH* for normalization. Values of control cells were set to 1. Shown as mean fold change versus control + SEM, *n* = 3. Two-way ANOVA with Bonferroni post hoc test is shown. *** *p* < 0.001, silenced versus control cells, and ^###^ *p* < 0.001, *MAX*-silenced versus *BMAL1*-silenced cells.

Notably, MAX was also detected on the promoters of *NR1D1* and *NR1D2* (Figure 4A), which were not significantly affected by the knockdown of *MAX*. Because BMAL1 and NR1Ds regulate each other, forming a feedback loop in the circadian signaling network [5], it is conceivable that transcriptional de-repression of NR1D1/NR1D2 in *MAX*-silenced MDA-MB-231 was not observed because of the resulting compensatory lowering in BMAL1 levels. Consistent with this hypothesis, the knockdown of *MAX* in *BMAL1*-silenced cells significantly increased both *NR1D1* and *NR1D2* transcription (Figure 4B).

Altogether, these data reveal that MAX can operate as a direct repressor of core clock genes.

### 2.4. MAX and BMAL1 Regulate the Expression of Common Transcripts

**Figure 5:**
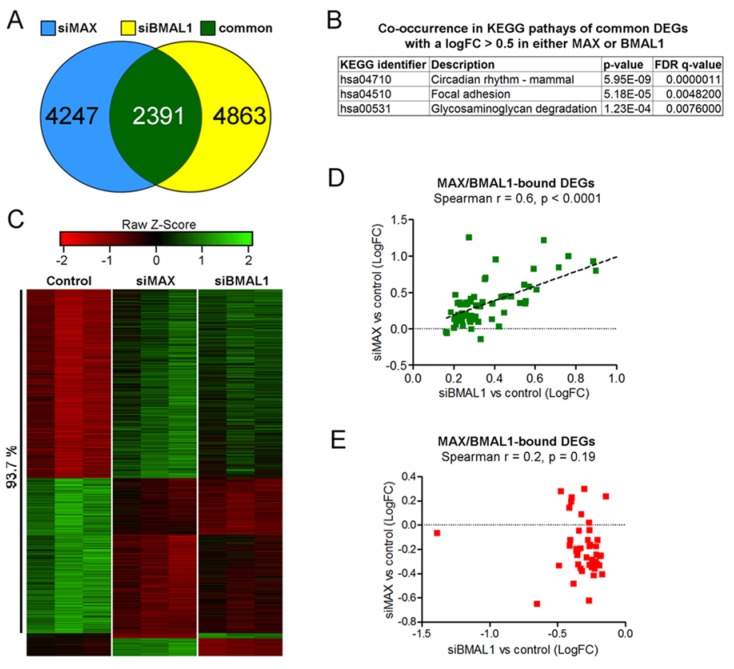
MAX and BMAL1 regulates the expression of common transcripts. (**A**) Venn diagram for differentially expressed genes (DEGs) in MDA-MB-231 cells with knocked down *MAX* (siMAX) or *BMAL1* (siBMAL1). (**B**) Co-occurrence in KEGG pathways of the set of common siMAX and siBMAL1 DEGs with an absolute logFC >0.5 upon the knockdown of either *MAX* or *BMAL1*. (**C**) Clustered heat map of triplicate normalized counts from common siMAX:siBMAL1 DEGs. The percentage of genes coherently altered in both *MAX*- and *BMAL*-silenced cells compared with control is shown. (**D**) Significant correlation between MAX/BMAL1-bound genes up-regulated in *BMAL1*-silenced and *MAX*-silenced cells. (**E***)* Lack of correlation between MAX/BMAL1-bound genes downregulated in *BMAL1*-silenced and *MAX*-silenced cells.

Genome-wide co-occurrence of BMAL1 and MAX on transcriptional regulatory regions suggests that these proteins might control the expression of common targets. To address this, we used a next-generation sequencing (NGS) approach for the identification of differentially expressed genes (DEGs) in MAX- or BMAL1-silenced MDA-MB-231 cells. Transcript assembly and quantification of RNA-sequencing reads identified 4863 and 4247 differentially expressed genes (DEGs) upon knockdown of either *BMAL1* or *MAX*, respectively (Supplementary Tables S3 and S4). The comparison of the two sets of genes revealed that 2391 of siBMAL1 DEGs (almost 50%) were also differentially expressed in MAX-silenced cells (Figure 5A; Supplementary Table S5). Within this subset, 662 transcripts showed a logarithmic fold change (LogFC) greater than 0.5. We thus analyzed their co-occurrence in KEGG pathways for evaluating the transcriptional signaling more affected by both MAX and BMAL1, using a false discovery rate (FDR) *q*-value <0.01 as a cut-off. Strikingly, this analysis identified the circadian rhythm as a highly significant pathway (*q* < 0.00001), together with focal adhesion and glycosaminoglycan degradation (Figure 5B). Consistent with our quantitative RT–PCR experiments, the list of transcripts co-regulated by MAX and BMAL1 included *period* and *cryptochrome* circadian repressor genes (Supplementary Table S5). In addition, another negative regulator of BMAL1/CLOCK-mediated transcription, *BHLHE41* (also known as *DEC2*) [40], resulted under the control of both MAX and BMAL1.

Notably, heat map and clustering analysis of DEGs in siMAX and siBMAL1 cells revealed that more than 90% of these genes (2241 out of 2391) were coherently altered in both conditions (i.e., their expression was altered in the same direction upon either MAX or BMAL1 silencing; Figure 5C). These results suggest that the overall effect of MAX reduction in silenced cells is a derepression of genes that are negatively regulated by the BMAL1-containing clock repressor complex. Supporting this hypothesis, LogFC values of BMAL1/MAX-bound genes upregulated by BMAL1-silencing positively correlated with their corresponding LogFC values in MAX-silenced cells (Figure 5D). In contrast, no significant correlation was observed between BMAL1/MAX-bound genes downregulated upon the knockdown of *BMAL1* (Figure 5E).

### 2.5. MAX Regulates Circadian Gene Expression

The above results suggest that MAX might have a direct role in circadian transcriptional regulation, thus contributing to the rhythmic oscillatory expression of clock target genes. This aspect can be investigated by synchronizing the circadian clock of cells with a proper stimulus, such as dexamethasone [41]. However, several reports indicated that a number of cancer cell lines, including MDA-MB-231, have a reduced circadian oscillation compared to non-cancerous cells such as MCF10A [42,43,44]. Indeed, real-time monitoring of MDA-MB-231 expressing a firefly luciferase reporter controlled by the *BMAL1* promoter synchronized with a dexamethasone stimulus showed a rhythmic oscillation only over a 48-h period (Supplementary Figure S4). Nonetheless, this oscillatory pattern was abolished in MDA-MB-231 reporter cells silenced for *BMAL1*, thus indicating its dependency on a master clock regulator. In addition, the knock down of *MAX* strongly reduced the amplitude of the observed oscillation (Supplementary Figure S4).

**Figure 6:**
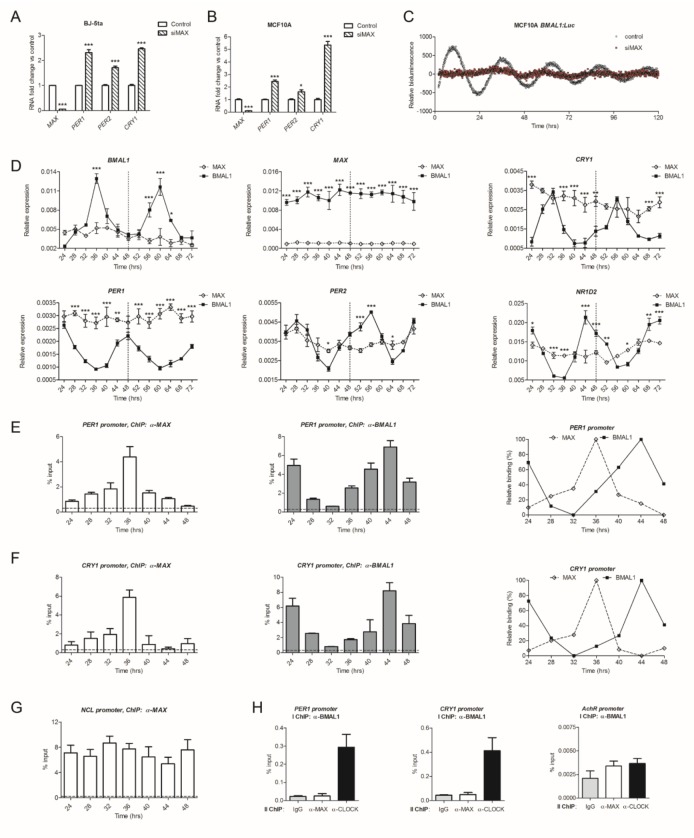
MAX is required for circadian gene expression. (**A**,**B**) Expression of *MAX*, *PER1*, *PER2*, *CRY1*, and *CRY2* genes upon MAX silencing in foreskin fibroblast BJ-5ta (**A**) and epithelial MCF10A (**B**) cell lines. Relative expression was determined by qRT–PCR using *GAPDH* for normalization. Values of control cells were set to 1. Shown as mean + SEM, *n* ≥ 3. * *p* < 0.05 and *** *p* < 0.001, two-way ANOVA with Bonferroni post hoc test, silencing versus control. (**C**) Bioluminescence counts from circadian synchronized MCF10A cells expressing a firefly luciferase reporter controlled by the circadian-responsive *BMAL1* promoter with knocked down *MAX* (siMAX, red dots). Cells transfected with a non-coding control was used as a control (control, black dots). Shown as baseline-subtracted luminescence data fitted to a sine wave. (**D**) Time-related expression over two circadian cycles of endogenous clock-controlled genes in siMAX and control synchronized MCF10A cells. Relative expression at the indicated dexamethasone post-treatment time points was determined by qRT–PCR using *GAPDH* for normalization. Shown as mean + SEM, *n* =3. * *p* < 0.05, ** *p* < 0.01 and *** *p* < 0.01, siBMAL1-silenced versus control cells, siMAX-silenced versus control cells, two-way ANOVA with Bonferroni post hoc test. (**E,F**) Recruitment of MAX and BMAL1 on the E-box-containing promoters of *PER1* (**E**) or *CRY1* (**F**) in MCF10A cells at the indicated post-synchronization time. Enrichment of MAX or BMAL1 was evaluated by quantitative PCR of immunoprecipitated DNA compared with input DNA (% of input). Immunoglobulin G (IgG) was used as a negative control. Normalized MAX and BMAL1 binding profiles are shown on the right panel for comparison (maximal and minimal values were normalized to 100 and 0, respectively). (**G**) Recruitment of MAX on the promoter of a non-circadian MAX target gene (*NCL*) at the indicated post-synchronization time. (**H**) The independent binding of MAX and BMAL1 to the clock-controlled promoters, *PER1* and *CRY1*, was evaluated by ChIP and Re-ChIP assays. Chromatin samples from non-synchronous MCF10A cells were first immunoprecipitated with an anti-BMAL1 antibody, and the recovered material was subsequently re-immunoprecipitated with IgG or with antibodies against MAX or CLOCK. A clock-independent promoter (*AchR*) was used as a negative control. Shown as mean + SEM, *n* = 2.

Because of the weak oscillation displayed by MDA-MB-231 cancer cells, we thus decided to evaluate whether MAX could control clock gene transcription in two non-cancerous human cell lines, foreskin fibroblast BJ-5ta and epithelial MCF10A. Similar to our observations in diverse cancer cell lines (Figure 1F), *MAX* silencing increased the levels of clock transcripts in both BJ-5ta and MCF10A (Figure 6A,B). We thus silenced *MAX* in MCF10A cells expressing a firefly luciferase reporter controlled by the *BMAL1* promoter and monitored luciferase activity after circadian synchronization by dexamethasone treatment. This analysis showed a rhythmic oscillation of luminescence in dexamethasone-treated control cells (goodness of fit = 98.3%, period = 26.23 h, and amplitude = 204.20; Figure 6C). Remarkably, this oscillation was markedly impaired upon the knockdown of *MAX* (Figure 6C) and no significant fit was observed for siMAX luminescence (goodness of fit = 17.3%).

To confirm and extend this analysis to endogenous clock genes, we then collected RNA samples from dexamethasone synchronized MFC10A cells every 4 h over two circadian cycles (from 24 to 72 h after dexamethasone treatment). Consistent with our luminescence analysis, *MAX*-silenced cells showed a reduced *BMAL1* oscillatory expression compared with control cells (Figure 6D). Furthermore, the reduction of MAX also blunted the rhythmic expression of the circadian genes *CRY1*, *PER1*, *PER2,* and *NR1D2*, which promoters were bound by MAX in our ChIP-seq and qChIP analyses. Of note, *PER1* and *CRY1* transcript levels were constitutively higher at all time-points in MAX-silenced synchronized cells, which is consistent with their elevated expression in non-synchronous MCF10A cells upon knockdown of *MAX*.

Notably, *MAX* transcripts did not show circadian variations in control cells, suggesting that the expression of MAX is independent from the clock machinery. Nonetheless, this result does not preclude that MAX might be recruited on the promoters of the clock genes in a time-dependent manner. We thus immunoprecipitated MAX or BMAL1 in chromatin samples from dexamethasone synchronized MCF10A cells every 4 h over a circadian cycle (from 24 to 48 h after dexamethasone treatment). In line with observations from mouse liver samples [45], BMAL1 showed a time-dependent recruitment on the promoters of the circadian genes *PER1* and *CRY1* (Figure 6E,F). Strikingly, MAX also bound rhythmically to these clock genes but with a different circadian pattern. Indeed, the maximal binding of BMAL1 was delayed by about 8 h compared to MAX binding (Figure 6E,F).

Of note, the recruitment of MAX on a non-circadian target, *NCL*, showed negligible time-related differences (Figure 6G), which is consistent with the absence of an oscillatory expression pattern of MAX in synchronized MCF10A cells.

We then evaluated whether MAX and BMAL1 concurrently bind to circadian promoters. We thus performed ChIP and Re-ChIP assays for determining whether MAX was present in a complex with BMAL1 on *PER1* or *CRY1* promoters. Chromatin samples from non-synchronous MCF10A cells were first immunoprecipitated with an anti-BMAL1 antibody, and the recovered material was subsequently re-immunoprecipitated with IgG or with antibodies against MAX or CLOCK.

As expected, the core clock complex formed by BMAL1 and CLOCK concurrently binds to both *PER1* and *CRY1* promoters, as indicated by a marked enrichment of the recovered DNA from samples re-immunoprecipitated with α-CLOCK compared to IgG (Figure 6H). Conversely, no enrichment was observed when α-BMAL1 immunoprecipitated samples were re-immunoprecipitated with α-MAX antibody.

Collectively, our results indicate that MAX regulates the circadian clock and temporally alternates with BMAL1 for the time-dependent binding to clock target promoters.

### 2.6. MAX Repressor Partner, MNT, Cooperates in MAX-Dependent Repression of Clock Genes

MAX can operate as either an activator or a repressor of transcription, depending on the interacting partner proteins [46,47]. Among these protein complexes, those formed by MAX and MNT actively repress the expression of genes containing E-box elements in their regulatory regions [48].

**Figure 7:**
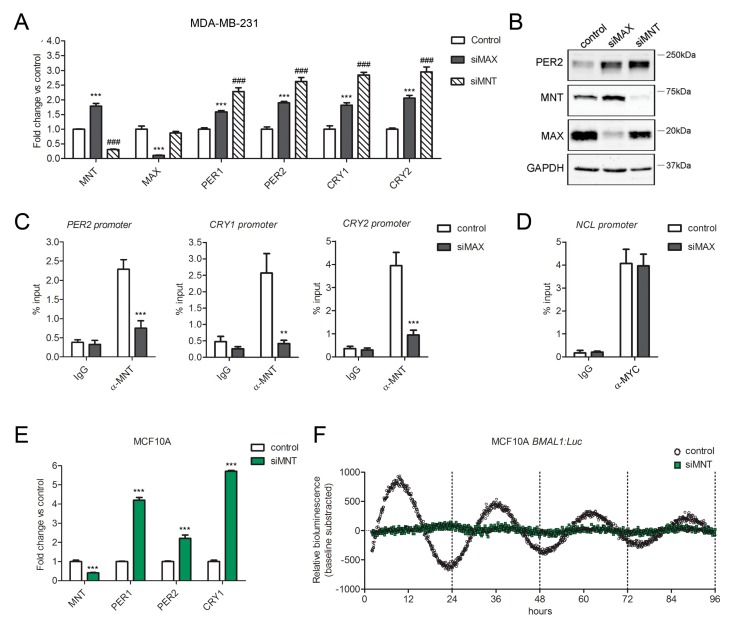
MAX repression of clock genes requires MNT. (**A**) Expression of clock genes *PER1*, *PER2*, *CRY1* and *CRY2* in MDA-MB-231 cells upon the knockdown of *MAX* (siMAX), *MNT* (siMNT). Relative expression was determined by qRT–PCR using *GAPDH* for normalization. Values of control cells were set to 1. Shown as mean + SEM, *n* ≥ 3. *** *p* < 0.001 and ^###^ *p* < 0.001, two-way ANOVA with Bonferroni post hoc test, siMAX or siMNT versus control cells, respectively. (**B**) Protein levels of PER2, MNT and MAX in MDA-MB-231 cells transfected with siRNA sequences against MAX (siMAX), MNT (siMNT) or a non-targeting control. GAPDH was used as a loading control. (**C**) Recruitment of MNT on the promoters of PER2, CRY1 and CRY2 in MAX-silenced (siMAX) and control MDA-MB-231 cells. Enrichment of MNT was evaluated by quantitative PCR of immunoprecipitated DNA compared with input DNA (% of input). Immunoglobulin G (IgG) was used as a negative control. Shown as mean + SEM, *n* =3. ** *p* < 0.01 and *** *p* < 0.001, two-way ANOVA with Bonferroni post hoc test, α-MNT precipitated DNA from siMAX versus control chromatin samples. (**D**) Chromatin samples from *C* (MAX-silenced and control) were immunoprecipitated with α-MYC. Enrichment of MYC on the MYC-target *NCL* promoter was evaluated by quantitative PCR of immunoprecipitated DNA compared with input DNA (% of input). Immunoglobulin G (IgG) was used as a negative control. Shown as mean + SEM, *n* =3. (**E**) Expression of clock genes *PER1*, *PER2*, *CRY1* in MCF10A cells knocked down for MNT (siMNT). Relative expression was determined by qRT–PCR using *GAPDH* for normalization. Values of control cells were set to 1. Shown as mean + SEM, *n* ≥ 3. * *p* < 0.05, ** *p* < 0.01 and *** *p* < 0.01, two-way ANOVA with Bonferroni post hoc test, silenced versus control cells. (**F**) Real-time bioluminescence oscillatory pattern in *MNT*-silenced (siMNT, green dots) and control (black dots) MCF10A cells expressing a firefly luciferase reporter controlled by the circadian-responsive *BMAL1* promoter. Shown as baseline-subtracted luminescence data fitted to a sine wave.

To investigate whether MNT might participate in MAX-mediated clock transcriptional repression, we evaluated the transcription of *PER1*, *PER2*, *CRY1*, and *CRY2* in MDA-MB-231 cells knocked down for MNT. This analysis revealed that MNT silencing significantly increased the expression of these clock genes (Figure 7A). Immunoblot analysis from MDA-MD-231 cells silenced for *MNT* also confirmed that the reduction of this MAX partner results in increased PER2 protein levels (Figure 7B).

Of note, the knockdown of MGA, which can also form repressive complexes with MAX, showed negligible differences in the expression of *PER1*, *PER2*, *CRY1*, and *CRY2* (not shown).

Suggesting that a repressive complex formed by MAX and MNT regulates clock gene expression, qChIP analysis from MAX-silenced MDA-MB-231 cells indicated the involvement of MAX in the recruitment of MNT on *PER2*, *CRY1,* and *CRY2* promoters. Indeed, the silencing of MAX significantly reduced the amount of MNT bound to clock gene promoters (Figure 7C). Notably, immunoprecipitation from the same MAX-silenced chromatin samples with an anti-MYC antibody revealed that the remaining MAX protein was still sufficient for a substantial binding of MYC on the promoter of NCL (Figure 7D), which is consistent with the fact that MAX reduction did not affect MYC dependent transcription in MDA-MB-231 cells.

Further indicating that MNT is also required for a proper circadian rhythm, the knockdown of this transcription factor significantly increased the expression of PER1, PER2, and CRY1 in non-synchronous MFC10A cells (Figure 7E) and strongly impaired the oscillation of the luciferase circadian reporter in *BMAL1*-luc MCF10A synchronized by dexamethasone treatment (Figure 7F).

## 3. Discussion

Our knockdown experiments indicate that MAX regulates the transcription of diverse genes belonging to the core clock machinery in both cancer and non-cancerous cell lines. Remarkably, knockdown of MAX in MDA-MB-231 was not sufficient to affect both the transcription of cell cycle-related MYC targets and proliferation, suggesting that the residual MAX protein still allowed for an MYC-dependent transcription of cell cycle genes. In line with this view, the recruitment of MYC on the NCL promoter was not affected by the knockdown of MAX in MDA-MB-231 cells. These data imply that MAX-mediated activity on clock genes can operate independently from the role of MAX as an MYC-associated factor. Supporting this concept, the knockdown of MYC produced negligible effects on the expression of core clock genes, and it did not alter the recruitment of MAX on *PER2*, *CRY1,* and *CRY2* promoters.

MAX actively represses numerous core clock genes by its direct binding to E-box-containing regions, as indicated by our ChIP-seq and qChIP analyses and by a lack of MAX-mediated derepression of a *PER2* promoter bearing a mutated E’-box sequence. Our genome-wide approaches further revealed that a reduction of MAX levels affects numerous BMAL1 regulated genes. Indicating that the MAX-mediated repression of clock genes plays a role in supporting a proper circadian rhythm, the reduction of MAX in MFC10A cells synchronized with dexamethasone blunted the oscillatory gene expression of both circadian reporters and endogenous clock-controlled genes.

Although MAX expression does not seem under the control of the clock machinery, MAX binding to the circadian promoters occurs in a time-dependent manner. Moreover, since MAX and BMAL1 do not concurrently bind the promoters of the clock genes (ChIP-Re-ChIP experiments), it would appear that MAX and BMAL1 contribute to two temporally alternating transcriptional complexes.

Notably, the core clock genes directly targeted and repressed by MAX include all the repressors belonging to both the primary (CRYs and PERs) and the accessory (NR1D1 and NR1D2) negative circadian feedback loops [5,20]. A proper stoichiometric balance between activators and repressors of E-boxes has been proposed as a crucial requirement for generating circadian rhythms [19,20]. Accordingly, MAX might have a function in preserving a balanced ratio between positive and negative elements of the molecular clock machinery.

We also identified MNT as a partner in MAX-mediated circadian regulation that is recruited on the promoters of clock core genes in a MAX-dependent manner. Our double knockdown experiments in MDA-MB-231 cells support a fundamental role of MNT in the repression of the molecular clock by MAX. In addition, MNT silencing strongly impaired circadian oscillation in both MDA-MB-231 and MCF10A cells. However, these results do not preclude the possibility that other heterodimerization partners of MAX [49] could contribute to the circadian clock in different cells or conditions (e.g., tissue development and differentiation), depending on the dynamics of MAX interactions [27].

Our data also imply that circadian alteration upon MYC overexpression [22,23] would depend on perturbation of physiological repression operated by the MAX/MNT complex. Indeed, it is well known that forced expression of MYC can antagonize with MNT for MAX binding to form a MAX/MYC activator complex [27,50]. Consistent with this view, there were increased MYC levels in U2OS upregulated clock genes, which promoters showed a direct recruitment of MAX and MNT in our ChIP experiments (i.e., *PERs*, *CRYs,* and *REV-ERBs*) [22].

The presented data also indicate that alteration of the MAX transcriptional network may contribute to circadian dysfunctions observed in several pathological contexts. For instance, the downregulation of MNT has been recently proposed as a important functional event for the hypoxia response in a wide variety of injury and disease settings [51], and severe consequences caused by acute hypoxia have been correlated with defects in circadian rhythms [52,53,54]. Although diverse proteins, such as HIF1A and mTOR, appear to interfere with the clock transcriptional regulation in low oxygen conditions [55,56], our data suggest that perturbation of MAX/MNT complexes might provide an essential contribution to hypoxia-induced chronodisruption.

In addition, genomic inactivation of MAX has been recently associated with an MYC-independent progression to malignancy of gastrointestinal stromal tumors [57], and future studies might reveal the contribution of the clock function of MAX in its paradoxical tumor suppressor role.

## 4. Materials and Methods

### 4.1. Cell Culture

Human breast cancer MDA-MB-231, BT549, human skin cancer A375, human stomach cancer SNU16, human embryonic kidney HEK-293 and HEK-293T cell lines (previously obtained from American Type Culture Collection (ATCC)) were grown in DMEM medium (Sigma, Darmstadt, Germanycatalog#D8537) supplemented with 4 mM L-glutamine (EuroClone, Milan, Italy catalog#ECB3000D), 10% fetal bovine serum (FBS) (Sigma, catalog#10001432) and 1× penicillin:streptomycin solution (Sigma, catalog#P4333).

Human liver cancer HEP-G2 cells (kindly provided by Istituto di Ricerche Farmacologiche “Mario Negri”, Milan, Italy) were grown in RPMI-1640 medium (EuroClone, catalog#ECB90006L) supplemented with 4 mM L-glutamine and 10% FBS.

Human epithelial MCF10A cell line (obtained from ATCC, Manassas, VA, USA) were maintained in a 1:1 mixture of DMEM and Ham’s F12 (Sigma, catalog#51651C) media supplemented with 20 ng/mL human epidermal growth factor (EGF), (Sigma, catalog#E9644), 2 mM L-glutamine, 5% horse serum (Sigma, catalog#H1270), 10 μg/mL human recombinant insulin (Sigma, catalog#I9278), 0.5 mg/mL hydrocortisone (Sigma, catalog#H6909), 100 ng/mL cholera toxin (Sigma, catalog#C8052), and 1X penicillin/streptomycin solution.

Human foreskin fibroblast BJ-5ta cell line (ATCC, catalog#CRL-4001) were maintained in a 4:1 mixture of DMEM and 199 (Sigma, catalog#M3769) media supplemented with 10% FBS, 2 mM L-glutamine and 1× penicillin/streptomycin solution.

All cell lines were maintained at 37  °C in a humidified atmosphere with 5% CO_2_.

### 4.2. siRNA Transfection

For RNAi experiments, 30 nM siRNA sequences against *MAX*, *MYC*, *BMAL1*, *CRY1*, *CRY2*, and *MNT* were reverse-transfected with DharmaFect 1 Transfection reagent (Dharmacon, Lafayette, CO, USA catalog#T-2001-03) following the manufacturer’s protocol. As a control, cells were transfected with MISSION^®^ siRNA Universal Negative Control #1 (Sigma, catalog#SIC001). Sequences adopted in siRNA experiments are shown in Supplementary Table S7.

### 4.3. Cell Proliferation Analysis

MDA-MB-231 cells were transfected with siRNA sequence against *BMAL1*, *MAX*, *MYC,* or a non-coding control. The number of cells was counted 24, 48, 72, and 96 h after transfection with a Countess II FL (Life Technologies, Carlsbad, CA, USA). Trypan blue staining was used to discriminate live and dead cells.

### 4.4. Quantitative RT–PCR

RNA and cDNA samples were prepared by Trizol (Life Technologies, catalog#15596018) extraction and retro-transcription with SuperScript ViloTM Master Mix (Invitrogen, catalog#11755-250) following the manufacturer’s protocol. Relative transcript expression levels were assessed by quantitative PCR with iTaqTM Universal SYBR Green Supermix (BioRad, Hercules, CA, USA catalog#172-5124) on a Via7 thermocycler (Invitrogen, Carlsbad, CA, USA). GAPDH transcripts were used for normalization. Primer sequences are listed in Supplementary Table S6.

### 4.5. RNA Sequencing

Total RNA samples were prepared by Trizol extraction followed by purification with a PureLink RNA kit (Invitrogen, catalog#12183018A). RNA integrity was examined using capillary electrophoresis on a BioAnalyzer 2100 (Agilent Technologies, Santa Clara, CA, USA). RNA libraries for sequencing were prepared with Illumina RNA TruSeq kit v2 (Illumina, San Diego, CA, USA catalog#15027084) following the manufacturer’s protocol. Libraries were sequenced using 50 base pairs paired-end on an Illumina HiSeq 2000 sequencer. RNA-Seq reads were aligned with tophat v.2.0.8 with -r 170 -p 8 --no-novel-juncs --no-novel-indels --librarytype fr-unstranded options [58].

### 4.6. Analysis of Differentially Expressed Genes

RNA-seq counts were used to determine differentially expressed genes with the DeSeq2 package (https://doi.org/10.1186/s13059-014-0550-8, 17/01/2020 included in the Galaxy web platform (usergalaxy.org). Differentially expressed genes (DEGs) were defined, adopting an adjust *p* < 0.001 as a statistical cut-off value. Lists of DEGs in BMAL1 and MAX-silenced cells, logarithmic fold change (LogFC), adjust *p*-values (adjP), and normalized counts are provided in Supplementary Tables S3 and S4. Analysis for co-occurrence in common KEGG pathways was the Molecular Signatures Database v6.2 package using a false discovery rate (FDR) <0.01. Output normalized counts from siBMAL1, siMAX, and control samples were used to generate heat map and clustering analysis of common DEGs in BMAL1 and MAX-silenced cells with Morpheus online software (https://software.broadinstitute.org/morpheus/ - 17/01/2020). Only genes with an absolute logarithmic fold change >0.5 were included in this analysis.

### 4.7. Quantitative Chromatin Immunoprecipitation

Chromatin immunoprecipitation (ChIP) experiments in MDA-MB-231-silenced cells were performed 48 h after reverse-transfection with siRNA sequences. ChIP experiments in MCF10A were performed on 80% confluent cells. Cells were cross-linked for 10 min with 1% formaldehyde (Sigma, catalog#F8775), neutralized with 125 mM glycine at pH 2.5 for 5 min, and washed twice in PBS. Cells were lysed with 0.5% SDS buffer containing protease inhibitors cocktail (Sigma, catalog# P8340), scraped and centrifuged at 1150× *g* for 10 min at 4 °C. Pellets were resuspended in 4 mL of ice-cold IP Buffer composed by a 2:1 micture of SDS buffer (100 mM NaCl, 50 mM Tris-Cl, pH8.1 EDTA, pH 8.0, 0.5% SDS) and Triton Dilution Buffer (100 mM Tris-Cl, pH 8.6, 100 mM NaCl, 5 mM EDTA, pH 8.0, 5% Triton X-100) containing protease inhibitor cocktail. Samples were sonicated with Branson Digital Sonifier (Danbury, USA) in 30 s bursts followed by 30 s of cooling on ice for a total sonication time of six minutes per sample. Chromatin was pre-cleared for 1 h at 4 °C with sepharose protein G beads (Life technologies, catalog# 101242) and subsequently precipitated overnight at 4 ⁰C with 4 µg of anti-BMAL1 (Protein Tech, Manchester, UK catalog# 14268-1-AP), 4 µg of anti-MAX (Bethyl Lab, Montgomery, TX, USA catalog#A302-866A), 4 µg of anti-MNT (Bethyl Lab, catalog#A303-626A), 4 µg of anti-MYC (Cell Signaling, Danvers, MA, USA catalog#13987S), and 4 µg of normal Rabbit IgG (Cell Signaling #2729S) as a negative control. DNA protein complexes were recovered with sepharose protein G beads overnight and washed twice sequentially with mixed micelle wash buffer (150 mM NaCl, 20 mM Tris-Cl, pH 8, 5 mM EDTA, 5% *w*/*v* sucrose, 1% Triton X-100, 0.2% SDS), LiCl/detergent buffer (0.5% Na-deoxycholate, 1 mM EDTA, 250 mM LiCl, 0.5% (*v*/*v*) NP-40, 10 mM Tris-Cl, pH 8.0), Buffer 500 (0.1% (*w*/*v*) deoxycholic acid, 1 mM EDTA, 50 mM HEPES, pH 7.5, 500 mM NaCl, 1% (*v*/*v*) Triton X-100), and TE buffer (10 mM Tris-Cl, 1 mM EDTA, pH 8.0). Beads were further supended in TE-S buffer (TE buffer, 2% SDS) and treated with RNAse A for 30 min at 37 °C. Cross-linking was reverted by overnight incubation at 65 °C in TE-S containing 0.4 mg/mL proteinase K. In parallel, inputs were treated in the same way. Immunoprecipitated and input DNA was purified using PCR purification kit (QIAGEN, Hilden, Germany catalog#28106) using 60 µL of buffer T (10 mM Tris-Cl, pH 8.0). Quantitative PCR was performed by using iTaqTM Universal SYBR Green Supermix. Primer sequences are listed in Supplementary Table S6. Promoter occupancy was calculated as percent of input using the following formula: (2^-(CT_ChIP_-CT_input_)) × (Input dilution factor).

### 4.8. Chromatin Immunoprecipitation Sequencing

For ChIP sequencing, immunoprecipitated DNA from MDA-MB-231 chromatin samples was obtained with the protocol described for quantitavie ChIP. Input and immunoprecipitated DNA (1–10 ng) were blunt-ended and phosphorylated, and a single ‘A’ nucleotide was added to the 3’ ends of the fragments in preparation for ligation to an adapter that has a single-base ‘T’ overhang. The ligation products was purified and accurately size-selected by agencourt AMPure XP beads (Beckman Coulter, Brea, CA, USA catalog# A63881). Purified DNA was PCR-amplified to enrich for fragments that have adapters on both ends. All the steps were performed on automation instrument, Biomek FX by Beckman Coulter. The final purified product was then quantitated prior to cluster generation on Bioanalyzer 2100. Libraries with distinct adapter indexes were multiplexed (1/5 libraries per lane) and after cluster generation on FlowCell were sequenced for 50 bases in the single read mode on a HiSeq 2000 sequencer.

### 4.9. ChIPseq Data Analysis

Alignments of reads and peak calling were performed using HTS-flow [59]. Brefly, ChIP-Seq reads were aligned on hg19 human genome assembly using BWA v.0.6.2 [60] and peaks were called with MACS2 v.2.0.9 [61], using a *p*-value threshold of 10^−8^. The normalized reads count in genomic regions (rpm) and plots were obtained using custom R scripts [62]. Peaks of ChIP-Seq were considered to belong to a promoter if at least 1 bp of the peak was contained in the (+300; +300) interval from TSS (transcription start sites). Promoters were retrieved from UCSC using the TxDb.Mmusculus.UCSC.hg19.knownGene R package [63].

### 4.10. Immunoblotting

Protein samples were extracted in RIPA buffer as described previously [64]. Immunoblot were performed on 20 µg of protein extracts separated on 8%–15% polyacrylamide gels and transferred onto nitrocellulose membrane (GeHealthcare Life Science, Marlborough, MA, USA catalog# 10600001). Immunoblot signals were visualized by the chemiluminescent ECL Star substrate (Euroclone, catalog# EMP001005) using the ImageQuant LAS-4000 Chemiluminescence and Fluorescence Imaging System (Fujitsu Life Science, Tokyo, Japan). Densitometry analysis was performed with ImageJ software (Wayne Rasband, USA).

### 4.11. Generation of PER2 Promoter Reporter Plasmids

A synthetic 778 base-pair DNA fragment corresponding to the wild-type human *PER2* promoter (from 238288785 to 238289562 of *Homo sapiens* chromosome 2, GRCh38.p12 primary assembly, sequence ID: NC_000002.12) and a corresponding fragment containing the E’-box sequence CACGTT mutated in CCCCCC were synthetized by and cloned in pBluescript II KS (-) vector by GenScript (USA). Wild-type and mutated PER2 promoter sequences were then sub-cloned in pGreenFire1-mCMV (EF1α-neo) (System Biosciences, catalog# TR010PA-N) upstream the minimal CMV promoter driving the expression of the copGFP protein. The resulting E’-box-GFP and E’^mut^-box-GFP vectors were sequenced with a specific primers in reverse orientation respect with the *copGFP* gene (copGFP RV primer, 5′-GATGATCTTGTCGGTGAAGATCACG-3′) to confirm the correct cloning.

### 4.12. Generation of Reporter Cell Lines

BMAL1:luc (obtained from Addgene, Watertown, MA, USA plasmid# 46824), E’-box-GFP and E’mut-box-GFP lentiviral vectors were packed in HEK293T cells by co-transfection with pVSV-G and pSAX2 plasmids (obtained by addgene, plasmid# 8454 and plasmid# 12260). Supernatant were collected 48 h after transfection and concentrated by ultracentrifugation. For lentiviral infection, MDA-MB-231 and MCF10A cells were exposed to lentiviral particle for 48 h prior to be moved to a selective medium containing either 1 µg/mL of puromycin (Sigma-Aldrich, St. Louis, MO, USA catalog# P8833, BMAL1:luc) or 600 ug/mL of G418 (Euroclone S.p.A, catalog# ECM0015Z, E’-box-GFP and E’^mut^-box-GFP). Single cell clones were selected via limiting dilution, and the expression of luciferase and GFP reporters was confirmed by luminescence and fluorescent microscopy analyses, respectively.

### 4.13. Real-Time Bioluminescence Monitoring of Circadian Rhythm in Cultured Cells

MDA-MB-231 and MFC10A cells expressing the circadian reporter BMAL1:luc were transfected with siRNA against BMAL1, MAX, MNT or a non-coding control. Fourty-eight hours post transfection, cells were synchronized by a treatment with 500 nM dexamethasone (dex) for 2 h. After the replacement of dex-containing medium with a warm phenol red-free medium supplemented with 0.4 mM D-luciferin (Invitrogen, catalog#L2916), cells were placed into a real-time bioluminescence reader (LumiCycle36, Actimetrics, Wilmette, IL, USA) maintained at 37 °C in a humidified atmosphere with 5% CO_2_. Luminescence was recorded every 5 min over a 4–6-day period. To compare rhythmic patterns in-silenced cells, baseline-subtracted luminescence data were fitted to a sine wave using LumiCycle analysis software (Actimetrics, Wilmette, IL, USA). For circadian analysis in MCF10A, a medium free of horse serum and hydrocortisone was used during synchronization by dexamethasone treatment and luminescence monitoring.

### 4.14. Sequencing Data Availability

The datasets and computer code produced in this study are available at Gene Expression Omnibus (https://www.ncbi.nlm.nih.gov/geo/query/acc.cgi?acc=GSE127192), accession numbers GSE127192 and GSE127212.

### 4.15. Statistical Analysis

For qRT–PCR, qChIP and cell proliferation analyses, statistical significance between groups was calculated by two-way ANOVA associated with Bonferroni post-tests. The correlation between BMAL1/MAX-bound DEGs genes was evaluated using Spearman’s rank correlation test. These statistical analyses were performed using Prism6 software package. Significance values were *p* < 0.05 (*), *p* < 0.01 (**) and *p* < 0.001 (***). For the analysis of of DEGs, the Benjamini–Hochberg adjusted *p*-value (adjP) was calculated using the DeSeq2 package and was considered significant at adjP < 0.05. Co-occurrence analysis of DEGs in KEGG pathways was performed with the Molecular Signatures Database v6.2 package (Broad Institute, http://software.broadinstitute.org/gsea/msigdb/index.jspof) using a false discovery rate (FDR) *q*-value < 0.01 as a statistical cut-off.

## Supplementary Materials

Supplementary materials can be found at https://www.mdpi.com/1422-0067/21/7/2294/s1. Figure S1. (A) Expression of MYC protein in triple negative (MDA-MB-231, BT-549) and non-triple negative (BT-474, MCF7) breast cancer cell lines. Epithelial MCF10A was used as a representative non-cancerous epithelial cell line while stomach SNU16 was used as a representative MYC overexpressing cancer cell line. (B) Specificity of MAX silencing on the expression of clock core genes. Expression of the inidicated clock genes in MDA-MB-231 cells upon knockdown of MAX with three diverse and non-redundant siRNA sequences. A non-coding siRNA was used as control. Relative expression was determined by qRT-PCR using GAPDH for normalization. Values of control cells were set to 1. Shown as mean fold change versus control + SEM, *n* = 3. Figure S2. (A) Expression of clock genes (PER1, PER2, CRY2, and TEF) in MDA-MB-231 cells with knocked down CRY1 (siCRY1), MAX (siMAX), and both CRY1 and MAX (siCRY1&siMAX). A non-coding siRNA was used as control. Relative expression was determined by qRT-PCR using GAPDH for normalization. Values of control cells were set to 1. Shown as mean ± SEM, *n* ≥ 3. * *p* < 0.05. ** *p* < 0.01 and *** *p* < 0.001, two-way ANOVA with Bonferroni post hoc test, silencing versus control. ###P < 0.001, siCRY1&siMAX versus siCRY1. (B) Repression of PER2-promoter driven GFP by a CRY agonist, KL001 (5 μM), in reporter MDA-MB-231 cell lines bearing either a wild type (E’-box-GFP) or a mutated (E’mut-box-GFP) PER2 promoter sequence. Expression of the endogenous PER2 was used as a control of the responsiveness of the cells to KL001. Two-way ANOVA with Bonferroni post hoc test comparing KL001 versus vehicle. ** *p* < 0.01 and *** *p* < 0.001. Figure S3 Ontological annotation of BMAL1 bound regions by Genomic Regions Enrichment of Annotations Tool (GREAT) (http://great.stanford.edu/public/html/). Table S1. Genomic regions bound by MAX identified by ChIP-seq analysis from MDA-MB-231 cells (ChIP-Seq reads were aligned on hg19 human genome assembly); Table S2. Genomic regions bound by BMAL1 identified by ChIP-seq analysis from MDA-MB-231 cells (ChIP-Seq reads were aligned on hg19 human genome assembly). Table S3. Differentially Expressed Genes (DEGs) in BMAL1 silenced MDA-MB-231 cells compared with control. Mean normalized counts (Mean), log fold change difference (LogFC) and adjusted P values (adjP) are reported. Table S4. Differentially Expressed Genes (DEGs) in MAX silenced MDA-MB-231 cells compared with control. Mean normalized counts (Mean), log fold change difference (LogFC) and adjusted P values (adjP) are reported. Table S5. Genes significanlty affected by the knockdown of either MAX or BMAL1 (adjust *p* < 0.05). Log fold change difference (LogFC) in MAX-silenced and BMAL1-silenced cells compred with control is reporter. Normalized counts of triplicates from siMAX, siBMAL1 and control samples is also reported. These values were used for generating the heat map in Figure 4C. Table S6. Primers used for gene expression analysis. Table S7. siRNA sequences used in this study.

## Abbreviations

BMAL1	Brain and Muscle ARNT-Like 1
MAX	Myc-associated factor X
PER1/2	Period circadian protein homolog 1/2
CRY1/2	Cryptochrome Circadian Regulator1/2
CLOCK	Circadian Locomotor Output Cycles Kaput
NR1D1	Nuclear receptor subfamily 1, group D, member 1
NR1D2	Nuclear receptor subfamily 1, group D, member 2
CCG	Clock Controlled Genes
CDK4	Cyclin-dependent kinase 4
CDC25C	Cell Division Cycle 25C
RCF4	Replication complex factor4
NCL	Nucleolin
MCM2	Minichromosome Maintenance Complex Component 2
CDKN1A	Cyclin Dependent Kinase Inhibitor 1A
ChIP-seq	Chromatin Immuno-Precipitation sequencing
NGS	next generation sequencing
DEGs	differentially expressed genes
BHLHE41	Basic Helix-Loop-Helix Family Member E41
HIF1A	hypoxia-inducible factor-1 alpha
mTOR	mammalian target of rapamycin
ATCC	American Type Culture Collection
